# Crystal structure of the *MSMEG_4306* gene product from *Mycobacterium smegmatis*

**DOI:** 10.1107/S2053230X18002236

**Published:** 2018-02-26

**Authors:** Adarsh Kumar, Subramanian Karthikeyan

**Affiliations:** a CSIR – Institute of Microbial Technology, Council of Scientific and Industrial Research (CSIR), Sector 39A, Chandigarh 160 036, India

**Keywords:** MSMEG_4306, *Mycobacterium smegmatis*, X-ray crystallography, zinc SAD, Rv2229c, zinc ribbon, coiled-coil helix

## Abstract

The *MSMEG_4306* gene from *Mycobacterium smegmatis* encodes a protein of unknown function with 242 amino-acid residues that contains a conserved zinc-ribbon domain at its C-terminus. Here, the crystal structure of MSMEG_4306 determined by the single-wavelength anomalous dispersion method using just one zinc ion co-purified with the protein is reported. The crystal structure of MSMEG_4306 shows a coiled-coil helix domain in the N-terminal region and a zinc-ribbon domain in the C-terminal region. A structural similarity search against the Protein Data Bank using MSMEG_4306 as a query revealed two similar structures, namely CT398 from *Chlamydia trachomatis* and HP0958 from *Helicobacter pylori*, although they share only ∼15% sequence identity with MSMEG_4306. Based on comparative analysis, it is predicted that MSMEG_4306 may be involved in secretion systems, possibly by interacting with multiple proteins or nucleic acids.

## Abstract 2

This article reports the crystal structure of MSMEG_4306, a hypothetical protein from *Mycobacterium smegmatis*, determined by zinc SAD phasing.

## 1. Introduction   

The rapidly increasing availability of genome-sequencing data has resulted in the prediction of many protein sequences for which function cannot be assigned owing to a lack of sequence similarity to any protein with known function. These proteins are generally classified as either hypothetical or uncharacterized proteins. Nearly 50% of the proteins predicted in prokaryotic genomes are annotated as hypothetical proteins (Hanson *et al.*, 2010 ▸). The functions of these hypothetical proteins, if determined, can provide valuable insights into the growth, metabolism, infectivity and other physiological processes of bacteria. Since it is difficult to predict the functions of hypothetical proteins based on sequence analysis alone, structural studies have been employed to predict the functions of hypothetical proteins (Teh *et al.*, 2014 ▸; Shrivastava *et al.*, 2017 ▸; da Fonsêca *et al.*, 2012 ▸; Park *et al.*, 2012 ▸).

The *MSMEG_4306* gene in *Mycobacterium smegmatis* encodes a protein of unknown function, and its homologue *Rv2229c* in *M. tuberculosis* has been shown to be essential for survival (Sassetti *et al.*, 2003 ▸; Griffin *et al.*, 2011 ▸; Xu *et al.*, 2013 ▸). Amino-acid sequence analysis of *MSMEG_4306* does not indicate statistically significant similarity to any protein of known function. Therefore, to assign a structure-guided function for MSMEG_4306, we initiated the determination of its crystal structure. Sequence analysis of MSMEG_4306 (UniProt ID A0R095) suggests that its sequence homologues are almost exclusive to actinobacteria, with closely related sequences being present in the *Mycobacteria*, *Nocardia* and *Rhodococcus* genera (with an identity of 60% or above over the entire sequence length). Moreover, MSMEG_4306 and its homologues contain a domain that belongs to the zf-RING_7 Pfam family (PF02591; Marchler-Bauer *et al.*, 2017 ▸). A characteristic feature of the zf-RING_7 family is the presence of a C4-type zinc-ribbon domain with two pairs of cysteines in a C*XX*C-*X*_18–26_-C*XX*C (zinc-finger) motif that can bind to zinc ions. Also, proteins containing the zf-RING_7 domain are predicted to bind nucleotides/nucleic acids (Rigden, 2011 ▸), proteins or small molecules (Bouhouche *et al.*, 2000 ▸; Eisenhaber *et al.*, 2007 ▸; Klug, 2010 ▸; Markus & Morris, 2009 ▸; Matthews & Sunde, 2002 ▸). Here, we report the crystal structure of MSMEG_4306 initially determined by zinc SAD phasing at 2.8 Å resolution and subsequently extended to 2.6 Å resolution. The crystal structure of MSMEG_4306 reveals a long coiled-coil helical domain in its N-terminal region and a zinc-ribbon domain in its C-terminal region.

## 2. Materials and methods   

### 2.1. Cloning, expression and purification of MSMEG_4306   

A pair of oligonucleotides for use as forward (5′-ATA AGG AAT CAT ATG AAA GCG GAA GTA AGC-3′) and reverse (5′-ATA ATT GTC GAC TCA CTG CTT GAC CCG-3′) primers with NdeI and SalI restriction sites (underlined), respectively, were chemically synthesized (Integrated DNA Technologies, USA). The *MSMEG_4306* gene was amplified by polymerase chain reaction (PCR) using the designed primers and genomic DNA of *M. smegmatis* mc^2^155. The purified PCR product was digested with the NdeI and SalI restriction enzymes (Fermentas, USA) for 5 h at 37°C, ligated into pre-digested pET-28b vector and transformed into *Escherichia coli* DH5α competent cells for amplification. The transformant colonies were screened for the presence of the insert by isolating the plasmid using a Miniprep kit (Qiagen, Germany) and digesting the plasmid with restriction enzymes. In addition, the integration of *MSMEG_4306* into pET-28b was also verified by automated DNA sequencing.

A confirmed clone (*pET-28b-MSMEG_4306*) was transformed into *E. coli* BL21 (DE3) cells and plated on an LB agar–kanamycin (30 µg ml^−1^) plate. A single colony from the LB agar–kanamycin plate was used to inoculate the primary culture in LB medium supplemented with kanamycin (30 µg ml^−1^), which was allowed to grow overnight at 37°C at 200 rev min^−1^. A secondary culture was set up by adding inoculum from the primary culture to LB medium supplemented with kanamycin (30 µg ml^−1^) to a final concentration of 1%. The culture was grown at 37°C at 200 rev min^−1^. Once the culture reached an OD_600_ value of 0.6, it was induced with 1 m*M* IPTG and allowed to grow further overnight at 18°C and 200 rev min^−1^. The cells were harvested by centrifuging the culture at 6000*g* for 10 min at 4°C. The pellet was resuspended in 20 ml lysis buffer [50 m*M* Tris–HCl pH 8.0, 150 m*M* NaCl, 1 m*M* dithiothreitol (DTT)] and disrupted by sonication on ice using an ultrasonicator (Sonics, USA) with 20% amplitude and a 30 s on, 30 s off cycle for 30 min. The supernatant was passed through 2 ml Ni–NTA beads (Qiagen, Germany) packed in a polypropylene column (Qiagen, Germany) which was pre-equilibrated with lysis buffer. The protein-bound Ni–NTA column was washed with 25 ml lysis buffer followed by 50 ml 20 m*M* imidazole in lysis buffer. The protein was eluted using 20 ml lysis buffer containing 250 m*M* imidazole. The eluted protein was dialyzed overnight against 500 ml dialysis buffer (with the same composition as the lysis buffer) at 4°C using a 10 kDa molecular-weight cutoff membrane (Small Wonder-Lyzer, Excellion Innovations and Inventions Inc.). The dialysed protein was concentrated using 10 kDa cutoff membrane concentrators (Pall Corporation, UK). The concentrated protein was further purified by size-exclusion chromatography (SEC) using a pre-equilibrated Sephacryl S-200 column (GE, USA). The major eluted peak was collected and concentrated using a centrifugal concentrator. The peak was compared with a standard molecular-weight plot to determine the oligomerization status of MSMEG_4306. The standard molecular-weight plot was generated by running standard protein markers (Bio-Rad, USA) on the SEC column. The concentration of protein was estimated by the Bradford method (Bradford, 1976 ▸). The concentrated protein was stored at −80°C (after flash-freezing in liquid nitrogen) until further use.

### 2.2. Circular dichroism (CD) analysis of MSMEG_4306   

For CD studies, MSMEG_4306 (in 50 m*M* Tris–HCl pH 8, 150 m*M* NaCl, 1 m*M* DTT) was diluted to a concentration of 0.3 mg ml^−1^ using autoclaved Milli-Q water. For the blank, the buffer was also diluted in the same way. Far-UV spectra were collected for the protein sample using a Jasco-810 CD spectro­polarimeter flushed with nitrogen at a rate of 9–12 l min^−1^. A cuvette with a path length of 1 mm along with a metal spacer block of 9 mm was used in the experiment. The scan was performed in the wavelength range 250–190 nm. For the denaturation experiment, the scan was performed at 222 nm in the temperature range 25–95°C with the temperature increasing at a rate of 2°C min^−1^. Renaturation of the protein was performed from 95 to 25°C with the temperature increasing at a rate of 2°C min^−1^. The data were obtained in the form of raw ellipticity and were converted to mean residual ellipticity (MRE) for further analysis.

### 2.3. Crystallization, data collection and structure determination of MSMEG_4306   

Crystallization trials for the initial screening of MSMEG_4306 (at 10 mg ml^−1^ in a buffer consisting of 50 m*M* Tris–HCl pH 8.0, 150 m*M* NaCl, 1 m*M* DTT) were set up using commercial screening kits such as Index (Hampton Research, USA), The Classics Suite (Qiagen, USA) and PEG/Ion (Hampton Research, USA) in 96-well sitting-drop plates (Molecular Dimensions, USA). Each crystallization drop was set up with 1 µl protein solution and 1 µl reservoir solution and equilibrated against 60 µl reservoir solution. The crystallization plates were incubated at 20°C and observed periodically for the formation of crystals. Optimization of the crystallization conditions was performed by varying the buffer concentration, pH and cryoprotection condition as well as the drop volumes. Seeding was also used to initiate nucleation. Optimization was performed using 48-well sitting-drop MRC Plates (Molecular Dimensions, USA) as well as sitting-drop Clover Plates (Rigaku Reagents, USA). The crystals obtained were mounted on a nylon loop (Hampton Research, USA) and flash-cooled in liquid nitrogen before data collection.

Initially, X-ray diffraction data were collected from an MSMEG_4306 crystal at a wavelength corresponding to the Zn *K* edge using synchrotron radiation on the BM-14 beamline, ESRF, Grenoble, France (and are hereafter referred as the Zn-SAD data). A total of 360 images were collected from a single crystal at a wavelength of 1.28198 Å (peak energy 9.688 keV). Data processing was performed using *HKL*-2000 (Otwinowski & Minor, 1997 ▸) to a resolution of 2.8 Å. The scaled intensities were converted to structure factors using *CTRUNCATE* (French & Wilson, 1978 ▸) as implemented in *CCP*4 (Winn *et al.*, 2011 ▸). Using the scaled data, a partial structure solution was obtained using the automated *CRANK*2 pipeline (Skubák & Pannu, 2013 ▸) in *CCP*4. The structure was then refined using *REFMAC*5 (Murshudov *et al.*, 2011 ▸) from *CCP*4 and *phenix.refine* (Afonine *et al.*, 2012 ▸) from *PHENIX* (Adams *et al.*, 2010 ▸). The model was manually built using *Coot* (Emsley *et al.*, 2010 ▸).

A second X-ray diffraction data set was collected from an MSMEG_4306 crystal at the home source using Cu *K*α radiation (wavelength 1.5418 Å) and a MAR345 image-plate detector mounted on Rigaku MicroMax-007 HF rotating-anode X-ray generator. The data were collected at 100 K using an Oxford Cryostream. A total of 149 images were collected and the data were processed using *MOSFLM* (Battye *et al.*, 2011 ▸) to a resolution of 2.6 Å. The structure of MSMEG_4306 obtained by zinc SAD phasing was used as a template to solve the structure from the 2.6 Å resolution data by molecular replacement using *phenix.phaser* (McCoy *et al.*, 2007 ▸) from *PHENIX*. The refinement was performed using *phenix.refine* and the model was built manually using *Coot*. Figures were generated using *PyMOL* (DeLano, 2002 ▸). The sequence alignment was generated using *MAFFT* (Katoh & Standley, 2013 ▸), and *ESPript* (Robert & Gouet, 2014 ▸) was used to overlay the structural features of MSMEG_4306 on to the sequence alignment. Structural alignment was performed using *LSQMAN* (Kleywegt, 1996 ▸).

## 3. Results and discussion   

### 3.1. Characterization of MSMEG_4306   

**Figure 1:**
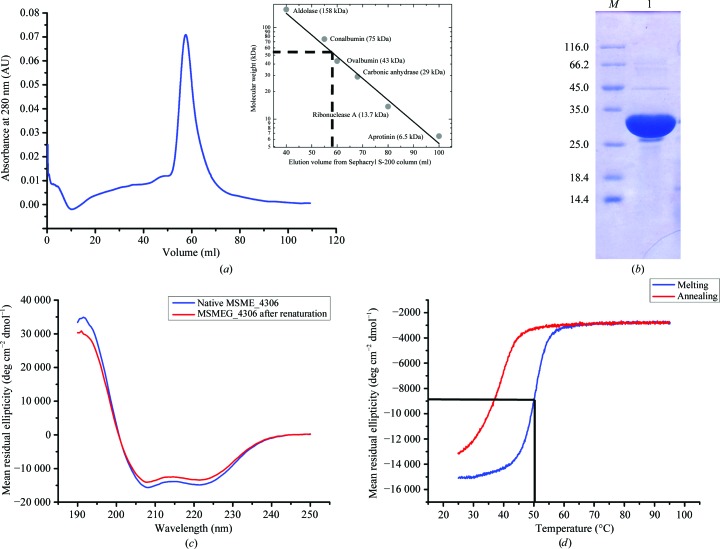
Characterization of MSMEG_4306. (*a*) Gel-filtration profile for MSMEG_4306. The standard molecular-weight plot is shown in the inset. (*b*) 15% SDS–PAGE showing purified protein. Lane *M* contains molecular-weight markers (labelled in kDa). (*c*) Far-UV CD scan for MSMEG_4306 before melting (blue) and after a melting and renaturation cycle (red). (*d*) Melting (blue) and renaturation (red) data for MSMEG_4306 at 222 nm wavelength.

The *MSMEG_4306* gene was cloned into pET-28b vector and expressed in *E. coli* BL21 (DE3) cells. The expressed MSMEG_4306 protein contained a 6×His tag and a TEV protease cleavage site at its N-terminus. The MSMEG_4306 protein was purified using Ni–NTA chromatography followed by size-exclusion chromatography. On comparing the standard molecular-weight elution profile, the position of the major eluted peak (∼59 ml) of MSMSEG_4306 in size-exclusion chromatography corresponds to a molecular weight of ∼50 kDa, suggesting that MSMEG_4306 might exist as a dimer in solution. The size-exclusion chromatography profile and the SDS–PAGE profile of the purified MSMEG_4306 protein are shown in Figs. 1 ▸(*a*) and 1 ▸(*b*), respectively.

To investigate the thermal stability of MSMEG_4306, CD studies were performed. Far-UV CD spectra were collected for MSMEG_4306 prior to melting (Fig. 1 ▸ *c*). The thermal stability of MSMEG_4306 was investigated by a melting experiment monitored at 222 nm in the temperature range 25–90°C (Fig. 1 ▸ *d*). Renaturation (Fig. 1 ▸ *d*) was also performed from 90 to 25°C and another far-UV scan was collected for MSMEG_4306 in order to check whether it refolds into its original state (Fig. 1 ▸ *c*). The CD experiments revealed that the thermal melting temperature (*T*_m_) of MSMEG_4306 was about 50°C. Interestingly, renatured MSMEG_4306 resulted in a near-identical scan to that of native MSMEG_4306, suggesting that the protein has an ability to refold on its own (Fig. 1 ▸ *c*).

### 3.2. Crystallization of MSMEG_4306   

During initial crystallization screening, small crystals of MSMEG_4306 appeared in multiple conditions within the period of a week. We optimized the crystallization conditions as well as the cryoprotection conditions and used seeding to improve the quality of the MSMEG_4306 crystals. The optimized crystallization condition was 20% PEG 8000, 5% PEG 2000 MME, 0.1 *M* Tris–HCl pH 8.5, 0.1 *M* magnesium formate. To obtain larger crystals, this crystallization condition was employed in a sitting-drop Clover Plate (Rigaku Reagents, USA) with a reservoir volume of 1000 µl and a total drop volume of 10 µl (5 µl reservoir solution + 5 µl protein solution). Diffraction-quality crystals were obtained in a week. For cryoprotection of the crystals, 2 µl each of 5, 10, 20, 30 and 35% glycerol in mother liquor were serially added to the drop at 2 min intervals. The crystal was then mounted on a loop and flash-cooled in liquid nitrogen before data collection.

### 3.3. Structure determination and quality of the model of MSMEG_4306   

A standard *BLAST* (Altschul *et al.*, 1990 ▸) search against the PDB using the MSMEG_4306 sequence as a query did not result in any significant structural homologue that could be used as a template for solving the structure by the molecular-replacement method. However, a *DELTA-BLAST* (domain enhanced lookup time accelerated *BLAST*) search (Boratyn *et al.*, 2012 ▸) revealed two crystal structures, namely those of HP0958 from *Helicobacter pylori* (Caly *et al.*, 2010 ▸) and CT398 from *Chlamydia trachomatis* (Barta *et al.*, 2015 ▸), as close homologues of MSMEG_4306, although they share only 15% identity with MSMEG_4306. Since the two homologues are likely to be too distantly related for successful molecular replacement, we decided to solve the structure of MSMEG_4306 by experimental phasing. Taking advantage of the presence of zinc ion in the MSMEG_4306 crystal, as confirmed by an X-ray fluorescence scan, an X-ray diffraction data set was collected at a wavelength corresponding to the Zn *K* edge using synchrotron radiation. The data set was indexed in space group *P*321, integrated and scaled to 2.8 Å resolution with an *R*_merge_ of 8.8% and an *R*_p.i.m._ of 2.0%. By assuming the presence of one molecule in the asymmetric unit, the crystal had a Matthews coefficient (Matthews, 1968 ▸) of 2.72 Å^3^ Da^−1^ with a solvent content of 55%. The structure of MSMEG_4306 was solved by zinc SAD phasing using the *CRANK*2 pipeline as implemented in *CCP*4. *CRANK*2 was able to locate one zinc ion and built a partial model for MSMEG_4306 with five fragments totalling 227 residues and identified the space group as *P*3_1_21. The partial model thus obtained was further refined using *REFMAC*5 and *phenix.refine*, and the model was built manually using *Coot*. During refinement of the structure, we were able to collect another data set for MSMEG_4306 from a slightly better looking crystal which diffracted X-rays to 2.6 Å resolution at the home source. The X-ray diffraction data from this MSMEG_4306 crystal were also indexed in space group *P*321, with an *R*_merge_ of 9.3% and an *R*_p.i.m._ of 3.4%. The structure of MSMEG_4306 was solved using the 2.6 Å resolution data set by molecular replacement in space group *P*3_1_21 using the Zn-SAD structure as a template. Further refinement was performed using *phenix.refine* and the model was built manually using *Coot*. Of the 242 amino acids in the sequence, we were able to build 235 residues along with one zinc ion, one glycerol molecule and 14 water molecules. Of these 235 residues, the side chains of 27 amino acids could not be traced in the electron density and were therefore only modelled up to the C^β^ atoms. Moreover, the residues from Leu86 to Ser90 as well as Lys241 and Gln242 could not be traced in the electron density and were therefore not included in the model. The final structure of MSMEG_4306 converged to an *R*_work_ of 23.1% and an *R*_free_ of 28.0%. The data-collection, processing and refinement statistics for both data sets are shown in Table 1 ▸.

### 3.4. Overall structure of MSMEG_4306   

**Figure 2:**
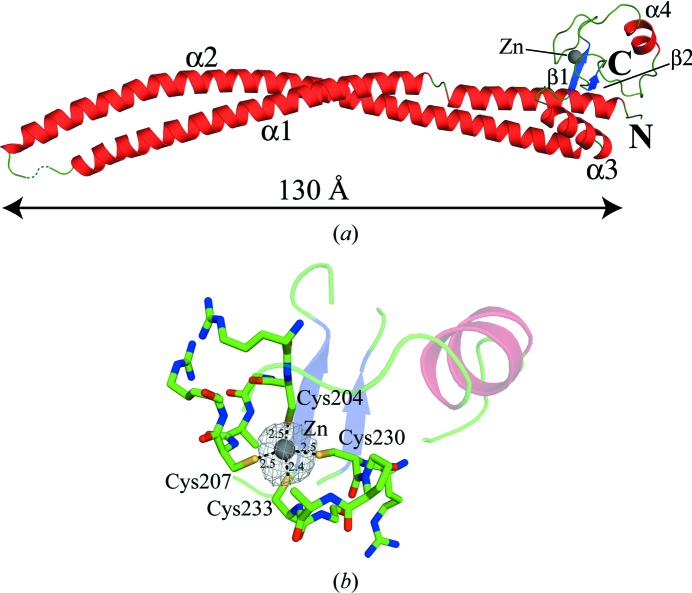
Structure of MSMEG_4306. (*a*) Cartoon diagram showing the C^α^ trace of the MSMEG_4306 structure. α-Helices are shown in red, β-strands are shown in blue and loops are shown in green. The zinc ion is shown as a grey sphere. (*b*) A close-up view of the zinc-ribbon domain and zinc ion coordination by cysteines. The two zinc knuckles are shown in green, with the C*XX*C motifs shown as sticks. The *F*_o_ − *F*_c_ map is shown as a grey mesh and is shown at a 3σ contour level. Distances are shown in Å and are marked as dashed lines.

The crystal structure of MSMEG_4306 (Fig. 2 ▸ *a*) revealed an elongated antiparallel coiled-coil helix domain (∼130 Å in length) in the N-terminal region and a globular zinc-ribbon domain in the C-terminal region. The N-terminal coiled-coil domain consists of two long α-helices. The first N-terminal α-helix (α1) is formed by Val5–Arg83, while α2 is formed by Val96–Ala176. The α1 and α2 helices are connected by a loop (for which density could not be traced) and interact with each other mostly by hydrophobic interactions through various leucines, isoleucines and valines present at the interface. In addition, a distortion present in the α1 helix between Lys29 and Ala32 causes the α1 helix to shift sideways about 6.5 Å, although the general direction of the helix remained intact. However, no such distortion was seen at the corresponding position in the α2 helix. The α2 helix is followed by the α3 helix (Asp179–Arg191), which acts as a connecting helix between the coiled-coil domain and the zinc-ribbon domain. The zinc-ribbon domain consists of one α-helix (α4) and two β-strands (β1, Ala196–Leu199; β2, Ile236–Leu239). The zinc ion in the zinc-ribbon domain is present in tetrahedral co­ordination with two pairs of cysteines (average bond length of 2.47 Å) present in two C*XX*C motifs separated by 22 amino acids (Fig. 2 ▸ *b*). Of these 22 residues, seven residues form an α-helix (α4, Glu215–Ala221) and the loops (zinc knuckles) bearing the two C*XX*C motifs are stabilized by two parallel β-strands. To check for potential oligomerization of MSMEG_4306, the crystal structure was analysed using the *PISA* (*Protein Interfaces, Surfaces and Assemblies* analysis) server (Krissinel & Henrick, 2007 ▸). *PISA* predicted a total of eight interfaces between symmetry-related molecules with relatively small interface areas, ranging from 28.9 to 567.1 Å^2^, indicating that the interactions between symmetry-related molecules may be owing to crystal packing and may not contribute to any biological oligomer formation. Although MSMEG_4306 eluted at a volume corresponding to a dimer in gel-filtration chromatography, this could be owing to the elongated dimensions of the protein. It is known that proteins with elongated dimensions can elute at a volume corresponding to double their actual size (Erickson, 2009 ▸), as elution is based on the hydrodynamic radius of the protein.

### 3.5. Comparison of MSMEG_4306 with other structures   

**Figure 3:**
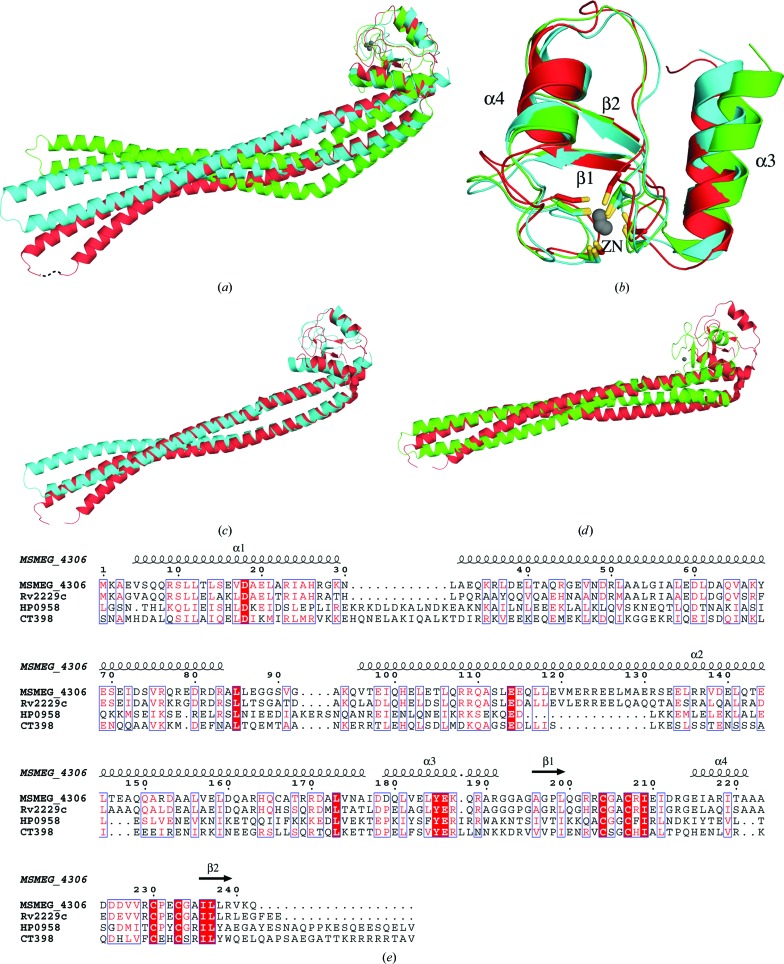
Comparison of MSMEG_4306 with structural homologues. (*a*) Overall structural alignment of MSMEG_4306 (red), CT398 (blue) and HP0958 (green) with the C-terminal domain as the reference. (*b*) Overall structural alignment of the C-terminal domains of MSMEG_4306 (red), CT398 (blue) and HP0958 (green). (*c*) Comparison between the structures of MSMEG_4306 (red) and CT398 (blue) showing deviation in the N-terminal coiled-coil helix. (*d*) Comparison between the structures of MSMEG_4306 (red) and HP0958 (green) showing deviation in the N-terminal coiled-coil helix. (*e*) Multiple sequence alignment of MSMEG_4306 with CT398 and HP0958. The secondary structure displayed at the top of the alignment is that of MSMEG_4306. Identical residues are shown in white with a red background, whereas similar residues are shown in red.

To investigate whether the MSMEG_4306 structure is similar to those of any other proteins, we compared it against all of the structures available in the PDB. In order to have a maximum chance of obtaining a similar structure, the MSMEG_4306 model was considered as three regions: the full-length protein (Met1–Gln242), the N-terminal domain (Met1–Arg191) and the C-terminal domain (Ala176–Gln242). The structures of these three regions were submitted to the *DALI* server (Holm & Rosenström, 2010 ▸) to search for similar structures in the PDB. In the case of the full-length structure, a total of 657 hits were obtained with *Z*-scores ranging from 15.9 to 2.0. However, on close inspection of the results we found that the majority of the hits were owing to the presence of long helices in the N-terminal domain and that only the top two hits were statistically significant. The top two hits were CT398 from *C. trachomatis* (PDB entry 4ilo, r.m.s.d. 3.8 Å, *Z*-score 15.9, 229 aligned C^α^ atoms; Barta *et al.*, 2015 ▸) and HP0958 from *H. pylori* (PDB entry 3na7, r.m.s.d. 7.7 Å, *Z*-score 15.6, 230 aligned C^α^ atoms; Caly *et al.*, 2010 ▸), which appeared to be strikingly similar to the structure of MSMEG_4306 (Fig. 3 ▸ *a*). Notably, a sequence-based search using MSMEG_4306 also indicated HP0958 and CT398 as distant homologues. Despite having poor sequence identity with MSMEG_4306 (16% for CT398 and 15% for HP0958), structural alignment between HP0958, CT398 and MSMEG_4306 (Figs. 3 ▸ *a* and 3 ▸ *b*) indicates a remarkable similarity in the zinc-ribbon domain, while the coiled-coil domains show variations in their orientations relative to each other. Although such structural variations may occur owing to different crystal packing (Eyal *et al.*, 2005 ▸; Rapp & Pollack, 2005 ▸), it is also possible that these variations are organism-specific and that they play a role in protein–protein interactions (Barta *et al.*, 2015 ▸). The search using just the N-terminal domain resulted in 846 hits, which comprised more or less the same proteins as those obtained with the full-length structure. The result for the C-terminal domain was interesting. Although several structures of zinc ribbons are known to exist in the PDB (PDB entries 1i50, 1adu, 1fre, 1dx8 *etc*.), *DALI* picked only three hits; the top hit was HP0958 from *H. pylori* (Caly *et al.*, 2010 ▸; r.m.s.d. 2.5 Å, *Z*-score 6.4, 63 aligned C^α^ atoms) and the other two hits were the chains of CT398 from *C. trachomatis* (Barta *et al.*, 2015 ▸; r.m.s.d. 2.3 Å, *Z*-score 5.9, 63 aligned C^α^ atoms). An alignment of the C-terminal domain of MSMEG_4306 with the HP0958 and CT398 structures is shown in Fig. 3 ▸(*b*). Similarly, structural alignments of the N-terminal domain of MSMEG_4306 with the N-terminal domains of CT398 and HP0958 are shown in Figs. 3 ▸(*c*) and 3 ▸(*d*), respectively. A multiple sequence alignment of MSMEG_4306 with Rv2229c, CT398 and HP0958 is shown in Fig. 3 ▸(*e*).

### 3.6. Prediction of potential biological function for MSMEG_4306   

Since a biological function for MSMEG_4306 could not be assigned based on its sequence alone, we explored whether the structure of MSMEG_4306 might guide us in assigning it a function. A comparative structural analysis of MSMEG_4306 and similar structures in the PDB revealed that a similar combination of an N-terminal coiled-coil helix domain and a C-terminal unique zinc-ribbon domain was only found in HP0958 and CT398. Both HP0958 and CT398 have been functionally characterized (Pereira & Hoover, 2005 ▸; Ryan *et al.*, 2005 ▸; Pereira *et al.*, 2011 ▸; Rain *et al.*, 2001 ▸; Douillard *et al.*, 2008 ▸; Barta *et al.*, 2015 ▸; Caly *et al.*, 2010 ▸). HP0958 is known to interact with RpoN (RNA polymerase, nitrogen-limitation N, σ^54^) and FliH (flagellar protein export apparatus protein, an ATPase regulator) (Rain *et al.*, 2001 ▸). Also, HP0958 enables the normal accumulation of RpoN by preventing its rapid turnover, thus acting as a chaperone for RpoN (Pereira & Hoover, 2005 ▸). Hence, the HP0958 gene affects most of the RpoN-dependent genes by modulating the stability of RpoN (Douillard *et al.*, 2008 ▸) and it has also been demonstrated to be essential for the motility of *H. pylori* (Ryan *et al.*, 2005 ▸). In fact, HP0958 has been shown to bind *flaA* mRNA and is predicted to work along with FliH to direct the *flaA* transcript for coupled translation and secretion of the FlaA protein (flagellar outer sheath protein; Douillard *et al.*, 2008 ▸). It has also been demonstrated that the *flaA* transcript binds to the zinc-ribbon domain of HP0958 (Douillard *et al.*, 2008 ▸; Pereira *et al.*, 2011 ▸). Thus, in *H. pylori*, HP0958 seems to be part of the flagellar-type III secretion system (T3SS). On the other hand, *C. trachomatis* has a complete set of nonflagellar type III secretion system (NF-T3SS) genes, but also encodes many homologues of flagellar T3SS despite the absence of flagella (Barta *et al.*, 2015 ▸). Just like HP0958, CT398 also shows binding to RpoN and FliH (Barta *et al.*, 2015 ▸). In addition, it has been shown to bind to CdsL, an ATPase regulator (Barta *et al.*, 2015 ▸). Barta and coworkers also demonstrated that only the N-terminal coiled coil is involved in interaction with RpoN, whereas interaction with FliH and CdsL is observed to involve both the coiled-coil and zinc-ribbon domains (Barta *et al.*, 2015 ▸). It has also been shown that CT398, RpoN and core RNA polymerase form a single complex in *C. trachomatis* (Barta *et al.*, 2015 ▸). Hence, CT398 has also been shown to be involved in T3SS events.

Given the prevalence of coiled-coil structures in protein–protein interactions (Mason & Arndt, 2004 ▸) as well as data from the CT398 and HP0958 structures, it appears that the N-terminal coiled-coil helix domain of MSMEG_4306 might also be involved in protein–protein interactions. The C-terminal zinc-ribbon domain, on the other hand, might be involved in both protein–protein and protein–RNA interactions. It is well known that mycobacteria lack the σ^54^ (RpoN) sigma factor (Manganelli *et al.*, 2004 ▸; Manganelli, 2014 ▸; Rodrigue *et al.*, 2006 ▸) as well as T3SS components (Feltcher *et al.*, 2010 ▸). However, a *DELTA-BLAST* search using the RpoN amino-acid sequence from *H. pylori* against the *M. tuberculosis* genome shows a sequence (locus SGC59090) with 29% identity (and 76% coverage), which is annotated as RNA polymerase factor σ^54^. Our attempts to identify homologues of the interacting partners of CT398 and HP0958 (FliH and CdsL) in mycobacteria using sequence-similarity searches did not yield any significant results. However, considering the near-identical domain organization in both HP0958 and CT398 to that of MSMEG_4306 and the near-identical function of HP0958 and CT398 despite low sequence identity (26%), we predict that MSMEG_4306 might be involved in secretion systems, possibly interacting with multiple entities (proteins or nucleic acids). In fact, the presence of Rv2229c (a close homologue of MSMEG_4306) has been reported in the cell-wall and membrane fractions of *M. tuberculosis* H37Rv (Mawuenyega *et al.*, 2005 ▸; Målen *et al.*, 2010 ▸; de Souza *et al.*, 2011 ▸), which further strengthens our belief in its involvement in secretion systems. However, further studies are required to confirm this.

## 4. Conclusion   

In this study, we have determined the crystal structure of MSMEG_4306 from *M. smegmatis*, which displays a long N-terminal coiled-coil helix and a C-terminal zinc-ribbon domain. Despite low sequence identity, MSMEG_4306 interestingly shows remarkable structural homology to HP0958 and CT398, both of which are part of type III secretion systems.

## Supplementary Material

PDB reference: MSMEG_4306, 2.8 Å resolution, determined by zinc SAD phasing, 5y05

PDB reference: 2.6 Å resolution, 5y06

## Figures and Tables

**Table 1:** Data-collection, processing and refinement details for MSMEG_4306 Values in parentheses are for the last resolution shell.

Data-collection wavelength (Å)	1.28198 (Zn-SAD)	1.5418
No. of crystals used	1	1
Resolution range for data processing (Å)	50.00–2.80 (2.90–2.80)	66.34–2.59 (2.71–2.59)
Space group	*P*3_1_21	*P*3_1_21
Unit-cell parameters (Å)	*a* = *b* = 77.137, *c* = 85.791	*a* = *b* = 76.60, *c* = 84.81
Total No. of reflections	161850	77310
Unique reflections (non-anomalous/anomalous)	7606/14091	9152
Average mosaicity (°)	0.90	1.07
Multiplicity	21.2 (18.8)	8.4 (7.9)
Overall 〈*I*/σ(*I*)〉	47.45 (1.93)	16.4 (3.5)
Completeness (%)	100.0 (100.0)	98.2 (85.1)
*R*_merge_ † (%)	8.8 (143.9)	9.3 (62.1)
*R*_meas_ ‡ (%)	9.1 (147.8)	9.9 (66.4)
*R*_pi.m._ § (%)	2.0 (33.3)	3.4 (23.0)
CC_1/2_	0.966 (0.952)	0.99 (0.70)
Resolution range for refinement (Å)	38.57–2.80	66.34–2.61
No. of reflections used	7226	8637
No. of *R*_free_-flagged reflections	376	460
*R*_cryst_ ¶ (%)	25.81	24.02
*R*_free_ †† (%)	31.18	27.13
R.m.s.d.‡‡		
Bond lengths (Å)	0.001	0.001
Bond angles (°)	0.308	0.363
Ramachandran plot, residues in		
Most favoured region (%)	98.25	99.13
Additionally allowed region (%)	1.75	0.87
Wilson *B* factor (Å^2^)	87.3	51.0
Average *B* factor for protein atoms (Å^2^)	91.7	70.8

† *R*_merge_ = 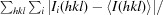 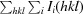, where *I*(*hkl*) is the intensity of reflection *hkl*.

‡ *R*_meas_ = 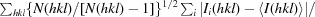 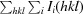.

§ *R*_p.i.m._ = 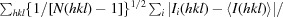 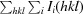.

¶ *R*_cryst_ = 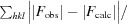 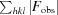.

†† *R*_free_ is the cross-validation *R* factor computed for the test set of 5% of unique reflections.

‡‡ Root-mean-square deviation.
